# Innate Immunomodulation in Food Animals: Evidence for Trained Immunity?

**DOI:** 10.3389/fimmu.2020.01099

**Published:** 2020-06-05

**Authors:** Kristen A. Byrne, Crystal L. Loving, Jodi L. McGill

**Affiliations:** ^1^Food Safety and Enteric Pathogens Research Unit, National Animal Disease Center, Agricultural Research Services, USDA, Ames, IA, United States; ^2^Department of Veterinary Microbiology and Preventive Medicine, Iowa State University, Ames, IA, United States

**Keywords:** trained innate immunity, veterinary species, disease resistance, beta-glucans, innate memory

## Abstract

Antimicrobial resistance (AMR) is a significant problem in health care, animal health, and food safety. To limit AMR, there is a need for alternatives to antibiotics to enhance disease resistance and support judicious antibiotic usage in animals and humans. Immunomodulation is a promising strategy to enhance disease resistance without antibiotics in food animals. One rapidly evolving field of immunomodulation is innate memory in which innate immune cells undergo epigenetic changes of chromatin remodeling and metabolic reprogramming upon a priming event that results in either enhanced or suppressed responsiveness to secondary stimuli (training or tolerance, respectively). Exposure to live agents such as bacille Calmette-Guerin (BCG) or microbe-derived products such as LPS or yeast cell wall ß-glucans can reprogram or “train” the innate immune system. Over the last decade, significant advancements increased our understanding of innate training in humans and rodent models, and strategies are being developed to specifically target or regulate innate memory. In veterinary species, the concept of enhancing the innate immune system is not new; however, there are few available studies which have purposefully investigated innate training as it has been defined in human literature. The development of targeted approaches to engage innate training in food animals, with the practical goal of enhancing the capacity to limit disease without the use of antibiotics, is an area which deserves attention. In this review, we provide an overview of innate immunomodulation and memory, and the mechanisms which regulate this long-term functional reprogramming in other animals (e.g., humans, rodents). We focus on studies describing innate training, or similar phenomenon (often referred to as heterologous or non-specific protection), in cattle, sheep, goats, swine, poultry, and fish species; and discuss the potential benefits and shortcomings of engaging innate training for enhancing disease resistance.

## Innate Modulation

While various approaches are used to limit disease and antibiotic usage in agricultural animals, efficacious intervention strategies remain unavailable for many diseases. Immunomodulation is one approach to engage or prime (1) the host's own immune system to defend against infectious disease. Vaccines are effective immunomodulators, priming the adaptive immune system, and well-understood by infectious disease experts. However, less common or at least less frequently discussed is immunomodulation of the innate arm of the immune system for enhanced disease protection. A related, rapidly evolving field of immunomodulation is innate training, which relies on memory of the innate immune system (see Text Box).

Box 1 **Defining immunomodulation.**

**Immunomodulation**

Changes to the immune system after exposure to a substance or compound (i.e., agonist) that stimulates or suppresses the immune response. This review is focused on immunomodulation that alters the immune response to subsequent exposure with non-related (heterologous) immune agonist, not the priming agonist.

**Immunomodulation of adaptive immunity**: altered vaccination or natural exposure of an animal to pathogens or other foreign agents induces the generation of effector and memory T- and B-cells to provide long-term (multi-year to lifetime) protection against the foreign agent.

Exposure of an animal to a priming substance or compound, often a protein or protein-polysaccharide, such that subsequent exposure to similar compound results in cross-reactive response. dependent on B- and T-cells. anergy and tolerance are functions of non-repsonsiveness or suppressive responses by B- or T-cells

**Immunomodulation of innate immunity (innate memory)**: exposure of an animal or cells to a priming substance or compound (i.e, agonist), often a microbial-associated molecular pattern (MAMP), such that exposure to non-related immune agonist results in heightened (trained) or suppressed (tolerant) response. Observed in non-T, non-B cells, and primarily in myeloid lineage and NK cells. Mechanism includes epigenetic and metabolic reprograming of innate immune cells and progenitor cells. Duration of effect is not yet determined, but there is evidence for months to a few years.

Training: *enhanced* response to heterologous agonists

Tolerance: *decreased* response to heterologous agonists

While adaptive immune memory is well-understood at the cellular and molecular level, the innate system was not known to have memory, and concordantly disease prevention strategies primarily targeted the adaptive immune system (e.g., vaccination). However, the paradigm on innate memory has recently shifted, with substantial evidence indicating that innate immune cells functionally adapt after stimulation or microbial exposure. More specifically, circulating monocytes, monocyte-derived macrophages, and NK cells have altered secondary responses to various pathogens or microbe-associated molecular patterns (MAMPs) after an initial priming event with the same or different MAMP (1–4). *In vitro*, purified monocytes stimulated with specific innate agonists, such as β-glucan or live-attenuated tuberculosis vaccine [*Mycobacterium bovis* bacillus Calmette-Guerin (BCG)], and restimulated days later with a heterologous MAMP, had heightened responses compared to cells that were not primed with β-glucan or BCG [reviewed in (3)]. Thus, the adaptive immune system may not be the only consideration for development of disease intervention strategies for enhancing food animal health.

**Figure 1:**
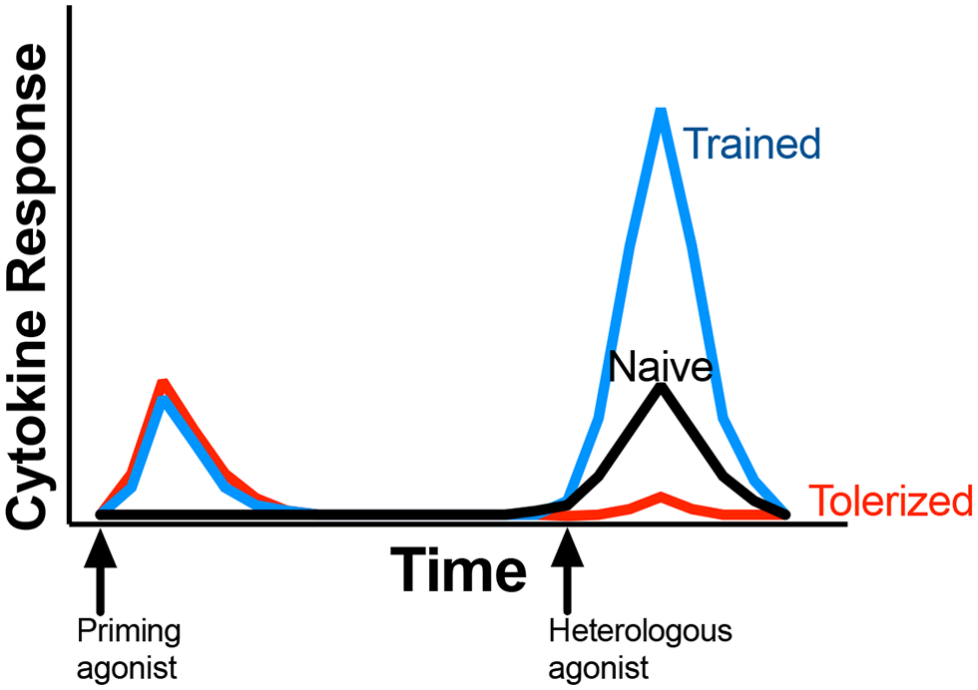
General outline of immune response associated with innate memory. Initial exposure to a priming agonist (red and blue lines) induces an innate response, typically measured as induction of proinflammatory cytokine production. After a rest period, subsequent stimulation of the same cells or animal with a heterologous agonist (commonly LPS or Pam_3_CSK_4_) results in tolerance or trained response, marked by reduced (red line) or enhanced (blue line) cytokine production when compared to naïve response (black line). Proinflammatory cytokines, including IL-1β, TNF-α, or IL-6 are the most common cytokines measured for innate memory. Priming and heterologous stimulation may all occur *in vitro* with primary cells; *in vivo*; or a combinantion of *in vivo* with *ex vivo* cell stimulation.

The sustained effect of trained innate immunity is dependent on epigenetic changes, chromatin remodeling, and basal metabolic shifts that occur in the cell after primary stimulation, with effects that can be long-lasting. Primary MAMP (5, 6) exposure leaves the cell in a “poised” state, or a state in which the cell is ready to respond to secondary insult or exposure. In a trained response, cells respond with increased production of effector molecules, including proinflammatory cytokines, upon secondary stimulation or exposure (Figure 1). The trained response differs from innate tolerance, in which poised cells respond with reduced production of effector molecules (Figure 1). While circulating immune cells such as monocytes and NK cells may exhibit a trained response, the lifespan of circulating cells is relatively short-lived (7) and the length of time cells exhibit a trained phenotype may concordantly be short-lived. However, epigenetic modification of bone marrow progenitor cells that become circulating effector cells (including monocytes and NK cells), underlies innate training *in vivo* and contributes to the longevity of innate training (8, 9). Experimental vaccination of humans with BCG enhances *in vitro* PBMC pro-inflammatory cytokine production upon stimulation with the MAMP lipopolysaccharide (LPS), even 12 months after BCG administration (1). Furthermore, epidemiological studies with human infants found that BCG vaccination is associated with non-specific (i.e., not related to BCG) protection. In other words, BCG vaccinated children had enhanced resistance to other diseases (5, 6), leading to increased overall survival and decreased incidences of morbidity. Importantly, the protective benefits were noted months to years after vaccination (10). Food animals are relatively short lived, and changes to innate immunity in early life may afford a protective effect against disease for the animals' entire lifespan.

In this paper, we briefly review important concepts from mouse, rabbit, and human literature, including mechanisms of innate memory, training, and tolerizing agents, and evidence for innate training in various cells types. However, the primary focus of this review is to summarize the available evidence for innate immunomodulation and memory in agricultural species including cattle, pigs, poultry, fish, and small ruminants.

## Review of Human and Animal Model Literature

### Immunomodulator Molecules

Bacterial endotoxin, or LPS, is the earliest described and best-known agonists capable of modulating the innate immune system (11). Priming of innate cells, most notably monocytes, with low doses of LPS, followed by homologous LPS restimulation resulted in a depressed inflammatory immune response (i.e., tolerance) (12, 13). However, Gregory Shwartzman described a phenomenon whereby rabbits intradermally injected with super low doses of gram-negative sterile culture filtrate had dermal necrosis at the site of injection following intravenous rechallenge with the same filtrate (14), suggesting the cells primed at the initial injection site responded to the secondary stimulation with a heightened response. The discovery of the “Shwartzman phenomenon” was followed by multiple studies highlighting the importance of dose, as low doses of LPS induced tolerance while super low doses resulted in heightened immune responses (15–18). Compounds besides LPS can induce a tolerant state in myeloid cells when restimulated with the same molecule (homologous tolerance). Additionally, priming with one agonist could induce tolerance in response to restimulation with a heterologous agonist, a process termed cross-tolerance (13, 19, 20). Zymosan, a particulate preparation of β-glucans, mannans, and proteins from *Saccharomyces cerevisiae* was one of the earliest known inducers of cross-tolerance (21, 22). Collectively, various molecules have been implicated in tolerance, and the dose upon primary exposure impacts the induction of tolerance.

As LPS is known as a classic tolerizing agent, the BCG vaccine is a highly described inducer of innate training. There are numerous reports on the non-specific effects of BCG vaccination of infants in countries that actively administer neonatal BCG, with significant reductions in non-tuberculosis diseases (5, 6, 23). In a set of particularly compelling studies, BCG vaccination of low-birth weight infants in Guinea-Bissau was associated with a nearly 50% reduction in mortality rates, primarily due to reductions in sepsis and respiratory infections (5, 6). Monocytes and NK cells from BCG-vaccinated adults and infants, compared to non-vaccinated cohorts, display increased expression of toll-like receptors and increased cytokine production in response to various pathogens and their products (e.g., *M. tuberculosis, Candida albicans, Staphylcoccus aureus*, LPS, and Pam_3_CSK_4_) (17–19). Mice vaccinated with BCG are protected from lethal *C. albicans* challenge via a mechanism that requires macrophages (24) and humans vaccinated with BCG show reduced viral titer when experimentally infected with the attenuated yellow fever virus vaccine strain (25). However, BCG-induced trained immunity did not protect mice against experimental influenza A infection (26). Early studies with *Mycobacterium tuberculosis* or BCG indicate enhanced resistance against subsequent disease may not be a universal phenomomen. While protection in mice against *Bacillus anthracis, Brucella suis, Staphylococcus aureus, Pasteurella pestis, Listeria monocytogenes*, and *Klebsiella pneumonia* has been noted (27–30), BCG treated mice are more sensitive to endotoxin and had similar mortality to untreated mice when challenged intravenously with a low dose of *Salmonella enteritis* (1 × 10^4^ CFU) (31). While not all encompassing, numerous reports indicate BCG administration in humans or rodent animals models enhances resistance to subsequent disease with increased immune responses to the secondary agent, a hallmark of innate training.

β-glucans, which activate innate cells via the Dectin-1 receptor or CR3, can also induce innate training. However, the source and type of β-glucan impacts the primary immune response and subsequent induction of innate memory. Human myeloid-lineage cells require cross-linkage of the Dectin-1 receptor and formation of a phagocytic synapse for downstream signaling (32). Soluble β-glucans, such as laminarin with (1–3)(1–6) linkages, can bind to the Dectin-1 receptor but are incapable of cross-linking multiple receptors and thereby fail to initiate an immune response (32, 33). As discussed above, zymosan induces tolerance in monocytes. It is a complex compound of β-glucan with highly branched (1–3)(1–6) linkages that, along with mannan and proteins, forms the cell wall. However, human and mouse monocytes primed with β-glucan from *C. albicans* exhibited a trained phenotype upon heterologous restimulation (17, 34–36). In fact, heat-inactivated *C. albicans* alone is sufficient to induce a trained state in human monocytes (34). β-glucans primarily induce innate training, but there are instances in which tolerance is induced, though the reason may not be related to dose, but receptor binding or signaling by additional cell wall components contained within the product.

There are many documented and perceived benefits associated with innate memory, but some potential drawbacks. While β-glucans and BCG are the most studied innate priming agonists other MAMP molecules, such as flagellin, muramyl dipeptide (MDP), polyinosinic-polycytidylic [Poly(I:C)] can induce innate training (1, 17). Innate training is the proposed mechanism for the non-specific benefits observed with certain vaccines, including the yellow fever vaccine, measles vaccine, vaccinia, and the influenza vaccine (37, 38). However, a heightened immune response to subsequent infections may result in enhanced pathology, an undesired effect. Indeed, trained immunity is hypothesized to contribute to autoimmune diseases (39, 40) and in a controlled experiment, patients vaccinated with BCG and later infected with malaria experienced earlier and more clinically severe symptoms than those not vaccinated with BCG (41). Thus, a deeper understanding of the implications associated with harnessing innate memory are warranted.

### Mechanisms and Cells in Trained Immunity

Trained immunity is based on epigenetic reprogramming in innate immune cells, which has been documented primarily in monocytes (35, 42). The epigenetic modifications lead to changes in gene expression and consequent protein production upon secondary stimulation. Chromatin modifications and changes in DNA accessibility are the central processes of epigenetic reprogramming associated with trained immunity. Histone modifications, such as increased methylation of the latent enhancer histone H3 at K4 (H3K4me1), reduced methylation of the repressor histone marks such as histone H3 at K9 (H3K9me3) and the most informative histone marker, increased acetylation at the poised/active enhancer mark (H3K27ac), are associated with a trained phenotype (1, 2, 35, 36). Other posttranscriptional regulatory mechanisms, such as microRNA (miRNA) modulation of mRNA levels, are involved in the regulation of the immune response (43). MicroRNA genes, as well as protein coding genes, can be regulated by histone modifications, and at the same time, miRNAs can directly and indirectly target effectors of the epigenetic machinery (44) and immune system mRNAs.

Changes to metabolic state are also noted in trained cells, and likely the result of epigenetic reprogramming whereby cells are primed to respond to secondary stimulation. Metabolic state is important for rapid release of intermediate substrates, such as nucleic and amino acids, necessary for the production of effector molecules (45). Innate training by *C. albicans* β-glucan is evidenced by an increase in basal glycolysis and a decrease in basal mitochondrial respiration, a measure of oxidative phosphorylation (Warburg effect) (46). The importance of glycolysis in β-glucan mediated innate training was noted in a clinical trial wherein β-glucan injection was administered to human volunteers, with a cohort also receiving metformin (a drug that prevents gluconeogenesis). Individuals on metformin did not exhibit enhanced *ex vivo* cytokine production following heterologous restimulation, which was in contrast to volunteers who received the β-glucan injection without prior metformin treatment (47). Thus, the availability and capacity to utilize glucose is critical in trained innate cells.

In addition to evidence of metabolic state impacting β-glucan induced training, BCG impacts cellular metabolism. BCG-treated cells have an increase in basal glycolysis as well as oxidative phosphorylation (48). Activation of the metabolic Akt/mTOR/HIF1 pathway is a critical feature of BCG-mediated trained immunity. Inhibition of glutamine or mTOR/glycolysis metabolism during *in vitro* training with BCG inhibited mRNA expression, and also prevented the epigenetic changes (H3K4me3 and H3K9me3) normally associated with BCG trained immunity (48). Collectively, the epigenetic and metabolic changes associated with BCG-induced training, or lack thereof, are linked. Understanding the mechanisms associated with training can provide a more targeted approach to modulate immune status.

Innate training and tolerance are well-described in monocytes and monocyte-derived macrophages, and to a lesser extent in NK cells (19, 34, 46, 49, 50). *In vitro* studies with purified monocytes and *in vivo* studies with severe combined immunodeficient (SCID) mice indicate T and B cells are not required for development of trained immunity (1). Epigenetic reprograming of myeloid progenitor cells leads to long lasting changes to emigrating monocytes, contributing to the longevity of innate training (8, 9). Tissue resident macrophages (e.g., Kupffer cells in the liver) are terminally-differentiated and do not rely on circulating monocytes for regeneration (51). It is unclear if tissue-resident macrophages can be trained, or if presence of trained myeloid lineage cells in tissue is the result of circulating monocytes migrating into a tissue. A study identifying alveolar macrophages with a trained phenotype showed CD8 T cells were required for induction of a trained state following a viral infection (52). In another study, reprogramming of tissue-resident macrophages occurred upon placement in new microenvironments, and cells may be driven into a trained or tolerant phenotype (53). Innate training is noted in other cell types, including dendritic cells (54, 55), non-immune cells such as mesenchymal and epithelial stem cells, and intestinal stromal cells (56). Additional research will be required to understand how alterations in the innate responsiveness of various cell types may contribute to disease resistance at the level of the organism.

## Innate Memory in Food Animals

Innate memory, defined as both training and tolerance, is well-described in human and rodent literature, and a mechanistic model of innate training as described above is beginning to be defined. However, there exists a paucity of information regarding innate memory in agriculture animals. Although broadly similar to human and rodent immune systems, there are important species-specific differences in the innate immune systems of individual food animals which can significantly impact the development of innate memory. For example, LPS dose plays a critical role in the induction of tolerance or training. However, it is difficult to draw parallels across species because of differences in LPS sensitivities. A very low dose of LPS can induce cellular and physiological changes in sheep, while a much higher dose is needed for a similar effect in chickens (57, 58). Thus, in the future, it will be critically important to assess the induction and effects of innate training in each individual species, ensuring that species-specific differences in innate immune function are fully acknowledged. As an impetus to encourage futher research, this review is focused on evidence for innate training in individual commercially important agricultural species and the potentiall benefits and limitations of innate training to enhance disease resistance.

### Cattle

To date, there are only a handful of reports detailing innate training as described by Netea et al. (3) in cattle. In one report, vaccination of 3- to 6- months old beef calves with heat-killed *M. bovis* resulted in an enhanced capacity for monocyte-derived macrophages from these animals to phagocytose and kill *M. bovis in vitro*. This effect was independent of cellular or humoral adaptive immune responses, and lasted up to 6 months after vaccination (59). Recent data from our group has shown that aerosol BCG vaccination induces a trained phenotype in circulating bovine monocytes. Specifically, monocytes isolated from BCG-vaccinated calves produce more proinflammatory cytokines in response to stimulation with LPS or Pam_3_CSK_4_ compared to cells from non-vaccinated, control calves (60). Thus, it is clear that the bovine innate immune system can be trained in a similar fashion as that of humans and rodents. Further analysis of the literature suggests other instances in which innate training may occur in cattle, although without experiments designed to specifically address the duration or mechanisms of innate memory, one must infer based upon the nature of the stimuli or resulting phenotype. In one report, immunization of cattle with an ultrasonicated lysate of *Corynebacterium cutis* had positive effects on morbidity and mortality in three different age groups of animals (61). Ten days old calves receiving the *C. cutis* lysate demonstrated a nearly 50% reduction in morbidity compared to controls due to enteric and respiratory diseases in the first 6 months of life. When pregnant cows were given *C. cutis* lysate in the final month of pregnancy, the resultant calves had a higher birth weight and greater weight gain in the first 3 months of life. Of the 23 control calves that were born, only 15 calves survived to 3 months of age, while 25 of 25 calves from the *C. cutis* immunized dams survived to the study endpoint (61). In a number of early studies, oral vaccination of calves using attenuated, live auxotrophic mutants of *Salmonella enteritidis* serovar Typhimirium (*S*. Typhimurium) resulted in homologous and heterologous protection against *S*. Typhimurium and *S*. Dublin (62–64). Protection was non-specific and T cell-independent, and endured for about 1 month after vaccination. Similar results were subsequently recapitulated in mouse models (65); and in fact, more recent results have shown that oral vaccination with live, attenuated *S*. Typhimurium induces sufficient non-specific protection to prevent lethal influenza virus infection in a mouse model (66). Thus, while the authors did not investigate the mechanisms of non-specific resistance in the calves, we speculate that the live *Salmonella* vaccine may have induced some form of innate memory.

Several recent commercial therapies have emerged with potential to enhance the bovine innate immune response during times of stress. One such DNA-based immunostimulant, marketed as the commercial product Zelnate™, can reduce lung-pathology scores in cattle experimentally challenged with *M. haemolytica* (67), and significantly reduce mortality in high-risk cattle after feedlot placement (68, 69). While the product's exact mechanism(s) of action is not well-defined, it is likely stimulating the immune system through pattern-recognition receptors such as TLR9 or the innate cytosolic DNA sensing c-GAS-STING pathway (70). It is unclear, however, if Zelnate™'s mechanism of action can be classified as innate memory. Product literature encourages the use of Zelnate immediately prior or within 24 h of a perceived stressful event. Given that a critical aspect of innate training is the duration of the effect, this form of immunomodulation may not fit the definition. Another immunomodulatory product, marketed as Amplimune™, is a mycobacterial cell wall fraction derived from the non-pathogenic *Mycobacterium phlei*. Amplimune™ non-specifically activates the innate immune system and can significantly reduce the incidence and severity of K99 *Escherichia coli* infection in newborn calves (71). It is currently marketed in the United States and Canada for this use. A recent study revealed that Amplimune™ also had significant beneficial effects in reducing the incidence and mortality associated with bovine respiratory disease in newly received, light-weight beef calves (72), suggesting it may have broader applications for ruminant health. Another commercial immunomodulator, Baypamun™, an inactivated preparation of Orf virus (Parapoxvirus ovis), was sold in Europe for several years for use in food animals and horses. Treatment with Baypamun™ immediately prior to, or in the early stages of infectious bovine rhinotracheitis infection was shown to significantly reduce clinical disease and virus shedding (73–76). Again, while it is evident that these commercial products have enhancing effects on the innate immune system, it is unclear if the immune system remains in a poised state for prolonged periods following treatment. More research will be required to determine if these products have the capacity to induce the long-term effects of innate memory, or simply a transient increase in innate activation.

The use of immunomodulatory feed compounds has grown with the increasing interest in alternatives to antibiotics. Many of these compounds are comprised of a mixture of whole yeast or yeast cell wall components. Several can promote innate immune functions, such as increasing phagocytic activity, increasing the generation of reactive oxygen species or restoring proinflammatory cytokine secretion to leukocytes from transition cows (77–82). The use of particular immunomodulatory feed additives can increase disease resistance in bovine. For example, supplementing with a *S. cerevisiae* fermentation product improves outcome of experimental *Salmonella* or *Cryptosporidium* challenges in preweaned calves (83–85); and reduces the size and number of liver abscesses in finishing beef steers, with efficacy comparable to standard in-feed antibiotic regimens (86). Yeast-supplemented cattle have reduced incidences of bovine respiratory disease during the receiving period (87, 88); while preweaned dairy calves receiving a yeast-based supplement have improved fecal scores and overall reductions in morbidity and mortality during the first 70 days of life. Given the capacity of the yeast cell wall component, β-glucan, to train the innate immune system in rodents and humans (34, 35, 46), it seems likely that at least some of the positive effects of such yeast-based additives on bovine health may be attributed to the induction of innate memory. More in-depth analyses of innate cell function and the specific epigenetic and metabolic alterations accompanying these changes will be required to determine if innate memory is a mechanism contributing to enhanced disease resistance.

### Sheep and Goats

Similar to the other species in this review, β-glucans are the most common immunomodulatory compounds investigated in sheep. Oral supplementation of β-(1-3)(1-6)-glucans to ewes has positive effects on reproductive performance, and on growth rate and body composition of the resultant lambs (89), potentially due to the positive effects of β-glucan supplementation on milk yield and milk composition in lactating ewes (89, 90). Monocytes and neutrophils isolated from lambs fed β-glucans have increased phagocytic and respiratory burst activities, and increased lysozyme activity (90, 91). Lactating ewes fed β-glucans have reduced somatic cell counts in milk (89), while an intramammary infusion of β-glucans resulted in selective recruitment of CD14^+^ monocytes/macrophages to the udder (92), potentially priming the animal to be more resistant to mastitis.

A recent series of studies has shown that the marine yeast, *Debaryomyces hansenii* and its cell wall, has the potential to train the innate immune system in newborn goats (93–96). *In vivo* supplementation of newborn goats with live *D. hansenii* induced upregulation of the genes encoding for TLR2, 4, and 6, IL-1β and TNF-α in circulating leukocytes, and resulted in increased respiratory burst, catalase and superoxide dismutase activity (94–96). The cell wall of *D. hansenii* is comprised primarily of (1-6)-branched (1-3)-β-D-glucan (96). *In vitro* training of goat monocytes with purified *D. hansenii* β-glucans results in increased expression of CD11b and the macrophage-associated gene F4/80, increased viability upon LPS challenge and increased phagocytic activity (93). *In vivo*, newborn kid goats supplemented with purified β-glucans from *D. hansenii* and subsequently challenged with LPS demonstrate increased plasma concentrations of IL-6, IL-1β, and TNF-α, and isolated leukocytes show increased respiratory burst activity and nitric oxide production (93).

BCG has not been widely studied in sheep. However, a few early studies showed that vaccination with BCG affords some resistance to infection with rift valley fever virus (97) and resistance to caseous lymphadenitis caused by *Corynebacterium pseudotuberculosis* (98). In the former study, a fraction of the BCG immunized sheep developed short fevers and viremia for only 24–48 h, while control sheep were viremic and febrile for up to 8 days after challenge. Sheep receiving two doses of BCG were completely protected from liver involvement due to rift valley fever infection (97). The latter study followed more than 500 head of sheep and used a model of natural *C. pseudotuberculosis* infection by seeding the herd with clinically infected animals. Over a period of 4 years, 99% of the lambs vaccinated with BCG were protected from development of caseious lymphadenitis (98). Thus, it appears that the innate immune systems of sheep and goats have the capacity to be trained, and the strategy holds significant potential for promoting disease resistance in small ruminants.

### Swine

β-glucans are readily used in pig production systems across the world with noted health benefits [reviewed in (99)]. Commerical in-feed products may be formulated with purified β-glucan from various sources (yeast, algae, fungi), or contain live yeast, yeast with fermentation products, or a semi-purified mix of cell-wall polysaccharides (mannans). Each product includes some amount of β-glucan, either purified or in the cell wall, which is presumably the ingredient responsible for noted changes in health status. Oral supplementation with β-glucans can improve weight gain, though not every trial indicates improved performance (100–103). Oral β-glucan can improve performance when low levels of aflatoxin are also present (104). Post-weaning diarrhea in pigs is caused by enterotoxigenic *E. coli* (ETEC), and antibiotics are commonly administered to limit ETEC. Oral β-glucan supplementation for the 2 weeks post-weaning decreased susceptibility to ETEC (105). Addition of yeast fermentation product to the diet decreases ETEC attachment to mucosa (106). However, frequency of diarrhea after ETEC challenge increases with a yeast-whole cell supplemented diet, although *E. coli* levels in feces do not increase (107). Interestingly, dietary β-glucan improves piglet health after rotavirus infection (108). However, inclusion of β-glucan in the diet increases susceptibility to intravenous *Streptococcus suis* challenge, even with increased performance measures (100). The authors hypothesize dietary β-glucan increases expression of IL-1R antagonist, which may enhance feed uptake by blocking IL-1R signaling, but enhances susceptibility to disease due to lack of necessary IL-1R signaling. Following LPS injection, pigs on a β-glucan diet have less TNF-α and IL-6 in the plasma (103), suggesting reduced responsiveness upon secondary stimulation. While β-glucan and related products are used in pig production systems, the mechanisms of improved health are not completely understood and various factors, including microbiota, age, and pathogen insult may impact outcomes.

Though β-glucan is most commonly administered by the oral route, changes in peripheral, as opposed to intestinal, immune status is often assessed. Multiple cell types have receptors for β-glucans in pigs, including myeloid progenitor cells in the bone marrow (22, 109, 110); and in mice, intravenous injected fluorescent labeled β-glucan is located in bone marrow macrophages and neutrophils (111). Thus, β-glucans may translocate from the intestine into the periphery to modulate progenitor cells, with peripheral impact. As noted above, levels of proflammatory cytokines in the sera are lower in response to LPS injection when pigs are fed a β-glucan supplemented diet (102, 103). Inclusion of whole yeast cells in the diet leads to shifts in circulating leukocyte populations (107, 112), though the impact of changes on disease resistance are unclear. Peripheral blood mononuclear cells produce less proinflammatory cytokine following LPS stimulation if pig diet is supplemented with β-glucan (103). While Dectin-1 and CR3 are expressed by different pig leukocytes (109), and intestinal dendritic cells may be the first to encounter dietary β-glucan (113), it's unclear how dietary supplementation alters responsiveness of peripheral immune cells to heterologous stimulation. Overall, dietary products containining yeast and/or β-glucan can modulate peripheral immune responses, but the longevity of the shifts and translation to innate memory warrant further investigation.

While BCG is a classically defined training agonist across multiple species, there are few reports on the impact of BCG administration on pig innate immunity. Coe et al. report BCG administration in pigs did not alter neutrophil function (114). In a recent study, pigs administered inactivated *Mycobacterium paratuberculosis* vaccine had enhanced pathology and inflammatory responses following *Actinobacillus pleuropneumoniae* challenge (115), suggesting a heightened secondary response indicative of innate training. BCG readily interacts with cells of the innate immune system; however, in pigs it's unclear if changes result in protection against heterologous infections. Pigs are a proposed model for tuberculosis research (116) and BCG vaccination in wild boar to limit *M. bovis* infection is proposed (117, 118). Given the conserved aspects of innate immunity across species, BCG is anticipated to induce innate memory in pigs, but has yet to be adequately demonstrated.

### Poultry

Of the food animals, commercial meat poultry (including chickens, turkeys, ducks, and quail) have some of the shortest times to market, with broiler chickens reaching market weights at an average of 47 days post hatch (119). While poultry breeds raised for egg production are longer-lived (1–2 years) (120), they have early life disease challenges and adaptive immune system limitations similar to meat poultry. Disease prevention via the adaptive immune system is controlled through vaccination against specific organisms and reaches full potential at around 3 weeks after vaccination (almost half a commercial broilers life span) (119, 121). Durning that 3 weeks span, maternal antibodies provide additional protection to the chick, but are dependent on multiple factors such as individual antibody titer levels and time post vaccination (122). Vaccination is relatively expensive with each vaccine providing protection against few pathogens. Contrasted to the adaptive immune response, the fast induction and breadth of innate memory presents a prime mechanism to reduce disease and foodborne organisms in poultry.

β-glucans are the most well-studied of the known innate memory immunostimulants in poultry (123, 124). However, due to the practical limitation of inoculating thousands of birds with β-glucans, stimulation with β-glucan occurs almost exclusively through the oral route via feed supplementation (125–129). Yeast are the most common form of dietary β-glucan as cereal β-glucans with (1–4)(1–6) linkages (i.e., barley and oat) are detrimental to poultry production due to reduced nutrient digestion and adsorption (130). In chicks, dietary supplementation with yeast β-glucans reduces *Salmonella* colonization of the cecum (131) and visceral organs (131, 132). Intermittent feeding of a β-glucan containing yeast product decreased the effects of transportation stress in turkey poults and tended to decrease colonization of the ceca with the foodborne pathogens, *Salmonella* and *Campylobacter* (129, 133). Interestingly, the same positive effect was not observed with continuous feeding of the yeast β-glucan product (129). No benefit of a β-glucan diet was observed when broiler chicks were challenged with *Eimeria* oocysts (128). While no studies in poultry have directly addressed the ability of β-glucans or other immunostimulants to induce trained immunity, β-glucan can alter the chicken immune system both *in vitro* and *in vivo*. Nitric oxide and IL-1, but not IL-6, production was increased in a chicken macrophage cell line following β-(1-3)(1-6)-glucan stimulation (134). The same study also detected increases in *ex vivo* macrophage phagocytic activity. Heterophil leukocyte function (phagocytosis, bactericidal killing, oxidative burst) is altered in yeast and β-glucan fed broiler chicks and turkey poults (125, 132, 133).

The production benefits (body weight, feed:gain ratios, feed consumption) of dietary β-glucans in poultry are less clear, as β-glucans and yeast products can enhance, reduce, or not change production parameters in chickens, turkey, or ducks (125, 127, 135). It is unclear if the conflicting results are due to different products (whole yeast, mannan oligosaccharides, purified β-glucan, etc.), source of yeast product (*Saccharomyces cerevisiae, Aureobasidium pullulan*, or other), relative dose of β-glucan, age of the animals, or some other factor. Huff et al. (135) suggest a potential mechanism for differences in production parameters, as they found in absence of *E. coli* challenge, chicks on control diets had higher body weights and feed:gain ratios than β-glucan fed chicks, but with challenge, the β-glucan chicks had higher production parameters. Dietary β-glucan is also associated with enhanced intestinal barrier functions (increased villus height/crypt depth ratio, number of goblet cells, and secretary IgA levels), but the authors did not determine if the effect was due to direct β-glucan stimulation of host immune cells or alterations in the gut microbial populations (131, 136).

*In ovo* injection of vaccines or immunostimulants represents an interesting way to alter the immune system of poultry before hatch and environmental exposure to pathogens. For the past 25 years, poultry producers have utilized *in ovo* technologies to safety and effectively vaccinate chicken, turkey, and quail embryos for common poultry diseases such as Marek's disease, infectious bursal disease (IBD), and coccidiosis (137). Recently, immunostimulants have come to the forefront of *in ovo* applications as a mechanism to non-specifically enhance the immune system of poultry before hatch. *In ovo* injection of resiquimod, a TLR7/8 agonist, at embryo day 18 increased MCR1L-B positive macrophages in the trachea, lungs, duodenum, and large intestine of chicks at hatch (138). Furthermore, the authors show that following infection with infectious laryngotracheitis virus (ILTV) 1 day post hatch, cloacal shedding of ILTV at 7 d post infection was significantly reduced in resiquimod injected embryos and that resiquimod treatment induced type 1 IFN activity in macrophages. Of the *in ovo* immunostimulants, CpG DNA is perhaps the most well-studied (139–141). Abdul-Cader et al. (140) show that CpG DNA delivered *in ovo* upregulates IL-1β expression and macrophage proportions in the lungs and these changes are associated with reduced ILTV induced mortality and weight loss in chicks. A 2018 study (139) with *in ovo* administration of CpG DNA reported reduced mortality and clinical scores from experimental *E. coli* infection of yolk sacs in day old chicks. Indeed, a commercial product Victrio®, is an *in ovo* DNA immunostimulant marketed to reduce mortality in embryonated eggs and chicks from *E. coli* and is shown to activate TLR21 on chicken macrophages and increase nitric oxide production (70). Overall, *in ovo* exposure to innate agonists altered immune responses to disease; however, it is unclear if the impact is the result of ongoing immune activation, or was the result of innate memory. The length of time from agonist exposure to challenge testing suggests innate memory may be at play, but targeted studies are warranted to clearly define the mechanism of protection.

### Fish

Unlike most other food animal species, review articles have been published summarizing the evidence for trained immunity in fish (142, 143) and we would direct readers to those sources for an in-depth review of innate training in various fish species. Since the 1990's, β-glucan from a variety of sources was studied or fed in commercial fisheries for its growth promoting and immunomodulating effects (144, 145). However, detailed analysis suggests the observed benefits are dependent on β-glucan source and dose, fish species, and age (145). Heterologous protection against bacterial challenge occurs after intraperitoneal or oral administration of β-glucans in multiple fish species ranging from Zebrafish (*Danio rerio*) (146) to Yellowtail (*Seriola quinqueradiata*) (147) to Orange spotted grouper (*Epinephelus coioides*) (148). Researchers observed lower mortality; increased oxidative burst, cytokine production, and lysozyme activity (143, 146, 147). The decreased mortality was observed up to 30 days after β-glucan feeding ceased (148). The length of effect after withdrawl suggests, similar to mammalian studies (34, 36, 123), dietary β-glucan treatment in fish induces epigenetic changes at the progenitor level allowing for sustained changes to innate immune cells. Fish express a higher diversity and variation of innate receptors that are both similar to and distinct from mammalian receptors (149). Dectin-1, a C-type lectin, is the primary myeloid receptor for β-glucans in mammals (32, 150–152); however, no corresponding β-glucan receptor has been identified in fish. Recently, Petit et al. (153) identified several potential candidate receptors for β-glucan in European common carp (*Cyprinus carpio carpio*). They also showed that, as with mammals, the C-type lectin pathway is involved in detection and signaling in response to β-glucan (153). This and other studies lay a foundation for mechanistic work in fish to determine the direct and long-lasting effect of β-glucan on the fish immune system.

Of the molecules known to stimulate innate memory (1, 123, 154), β-glucans are those most commonly used in aquaculture, but others, including mycobacteria, have been studied. Reviewed in Petit and Wiegertjes (142), studies show intraperitoneal injection with *Mycobacterium butyricum* enhances bactericidal activity up to 33 d post injection (155). A series of studies by Kato et al. (156–158) indicate injection with BCG enhances innate immune responses in multiple fish species and induces protection against challenge with *Nocardia seriolae* in Japanese flounder. Yellowtail (*Seriola quinqueradiata*) first exposed to one of several immunostimulants were protected against *Pasteurella piscicada* disease. Specifically, pre-exposure to Freund's complete adjuvant (CFA), which contains inactivated *M. bovis*, was found to be the most protective (147). Indeed, while enhanced survival and immune markers were observed with glucan pretreatment, the effect was markedly heightened with CFA treatment. The observed cross protection observed with these last studies are hallmarks of innate training and is strong evidence for innate memory in fish.

When reviewing literature published before innate memory was well-described, and in the absence of studies specificly designed to investigate the induction of heterologous protection independent of adaptive immune system, it can be difficult to assign innate memory as the mechanism for enhanced disease resistance. For example, Lorenzen et al. (159) observed cross-protection against viral hemorrhagic septicemia virus (VHSV) in rainbow trout inoculated with a plasmid DNA encoding the viral glycoprotein from an unrelated virus. Mortality was decreased in plasmid inoculated fish when challenged with virus either 4 and 7 days after plasmid inoculation, but not at 60 or 84 days post inoculation. The limited window of protection suggests a mechanism independent of adaptive immunity. A later study of juvinal turbot (*Scophthalmus maximus*) inoculated with DNA plasmid encoding the VHSV envelope glycoprotein and challenged with unrelated virus also observed reduced mortailities in the plasmid inoculated fish (160). It's unclear if the protection was due to innate training, or just non-specific protection due to the primary reponse to plasmid DNA. Regarless, some immunomodulation occurred to enhance disease resistance. Mechanistic evidence of contemporary trained immunity (as described by human and rodent literature) remains to be described. As demand for fish increases, methods to enhance disease resistance in farmed fish without high cost and antibiotics is desired.

## Implementing Innate Modulation in Food Animal Agriculture

The primary objective of innate immunomodulation in food animals is to enhance the immune status of the animal, thus resisting disease to enhance animal welfare and production efficiency. Enhancing the animal's ability to resist disease could reduce the need for antibiotics and amount of feed required to get an animal to market weight. In most production systems there are clearly defined periods in which animals are known to be at high risk for infection. Universal to all production systems is the susceptibility of the neonatal or very young animal to disease (161). As maternal immunity wanes, and an infant's own adaptive immune system is inexperienced, there is a window of heightened vulnerability to disease. The adaptive immune system may not be fully matured in neonates, but the innate immune system is active and provides a key role in immune responses at this age [reviewed in (162)] making it a good target for enhanced protection. In humans, innate training mediated by BCG is effective for more than a year (124). While longevity of protection by innate training is important, it may also be important to initiate protection early in life. The time from birth to market for a particular species can range from weeks to years, for example broilers go to market at around 6 weeks of age, pigs at 6 months, and beef cattle at 2 years. Thus, an important consideration for harnessing innate training is length of protection but also how quickly innate training protection is evident in the animal. One recent review of antibiotic usage by pig producers in Belgium reported that more than 80% of all antibiotics are administered to piglets <10 weeks of age (163); and similar results were reported for North America and several other countries in Europe (164). An important consideration for harnessing innate training is length of protection but also how quickly innate training protection is induced to minimize disease during a high-risk period.

Calves are most susceptible to diarrheal diseases in the first 4 weeks of life, and then the risk for respiratory diseases increases as they mature to 2–6 months of age. Induction of innate training in the neonatal period would be expected to promote improved disease resistance through at least 6 months of age, when the “window of susceptibility” to infection is the greatest (165). Subsequent high-risk periods for beef cattle include the shipping and receiving period (first 50 days on feed), when cattle are weaned, trucked, co-mingled, and placed in the feedlot (166). Administration of BCG, β-glucan, or other known immunomodulator in the period prior to weaning and shipping could protect the animal through the receiving period. In support of this supposition, the commercial immunomodulators Zelnate™ and Amplimune™ have both shown some benefit when administered to calves immediately prior to or following placement in the feedlot (67–69, 72). Dairy cattle are known to go through a period of immunosuppression during the transition period (the 3 weeks prior to calving, through the 3 weeks after calving) leading to a sharp increase in the prevalence of infectious and metabolic diseases (167, 168). Exploiting the effects of innate training or tolerance during the periparturient period could have beneficial effects on cow health and performance.

As with pigs and cattle, fish and poultry are most susceptible to disease while they are very young. In fish, immunostimulants have been administered to newly hatched larvae directly via feed pellets, or indirectly via bath treatment (143, 144, 169–171). In poultry, as described in detail above, *in ovo* vaccination is industry standard and there exists both experimental and commercial (Victrio®) evidence of protection from disease with non-specific immunostimulants (70, 139, 140). As egg laying animals, immunomodulation can occur before hatch and before the fry or chick is exposed to a broader range of pathogens. Additionally, the passive transfer of antibodies from the mother to the progeny via the yolk in egg-laying species is well-described, but in fish, innate immune components have been shown to transfer from the dams to the oocytes and direct passage of immunostimulants from mother to young have also been described (172–174). One last benefit of innate modulation in both poultry and fish is the evidence of passage of transgenerational epigenetic changes to innate immune phenotypes and genes for both broiler chickens (175) and fish (176, 177). Combined with a dam's ability to produce large numbers of eggs, changes in epigenetic phenotypes (the hallmark of innate memory) could be inherited by the embroyos allowing for animals to hatch in a primed state to face bacterial or viral challenge. In some ways fish and poultry producers have an advantage beyond that of mammalian species to harness innate memory to prevent disease, and both industries have made strides to study and utilize these evolutionary advantages.

### Potential Pitfalls of Innate Training

Although harnessing innate training for enhancing disease resistance is appealing, particularly in the context of the known periods of susceptibility or immunosuppression described above, there are still potential pitfalls that warrant consideration. In humans and rodents, innate training can have deleterious effects in the context of chronic inflammatory conditions such as autoimmunity, atherosclerosis, and diabetes (3). In food producing animals, while heightened immune responses may be beneficial for pathogen clearance, an increased inflammatory response may lead to tissue damage. In the case of respiratory diseases, for example, dysregulated inflammatory responses are often implicated as causing more damage to the host than the pathogen itself (178–181). Further enhancing this innate inflammatory response may not be ideal. However, if innate training has the capacity to reduce shedding of the organism, its use may still provide significant benefit to the health of the herd by reducing the risk of disease transmission. The impact of innate training on tissue pathology, pathogen burden and overall outcome of disease will need to be carefully evaluated in the context of particular disease settings in order to determine the risk vs. reward of engaging innate memory.

In addition to the potential of enhancing pathology of a disease, harnessing the immune system for disease resistance may negatively impact production parameters. Activation of the immune system comes at a considerable metabolic cost to an organism (182–185) and becomes energy not spent on production of muscle or milk. Activation of cells for the synthesis and secretion of cytokines and acute phase proteins, and cellular proliferation all require glucose, amino acids, and energy. To ensure survival of the host, the integrity of the immune system is maintained above nearly all other biological functions, partitioning nutrients away from growth, reproduction, and lactation (182). Although there is currently little supporting evidence available, it is almost certain that induction and maintenance of the innate immune cells in a trained state comes at some catabolic cost to the animal. Glycolysis, the metabolic pathway favored by trained monocytes and macrophages (46, 47) is less efficient than oxidative phosphorylation; thus increasing the cost of per-cell energy use. In addition, the initial activation of the immune system to induce a trained state utilizes metabolic resources. Although there is significant benefit to the animal in limiting disease upon pathogen exposure, it is currently unknown if the energy costs of a trained immune system will negatively affect performance, or if this possible performance loss will outweigh the benefit of increased disease resistance. Additional studies focused on the efficacy of innate training for preventing disease in food animals, as well as the impacts of training on animal performance and growth, will be required to unravel these possibilities. Regardless, strategies that enhance disease resistance without antibiotics warrant consideration to limit the impacts of antimicrobial resistance.

## Summary and Conclusions

Few studies have directly examined trained immunity in food animal species. However, as discussed here, a plethora of evidence exists for a variety of immunostimulants to enhance non-specific, heterologous protection against bacterial and viral disease in cattle, swine, poultry, fish, and small ruminants. Innate memory presents an exciting opportunity to prevent or limit disease as well as reduce antibiotic use and AMR in agricultural animals. A number of opportunities exist for mechanistic studies to elucidate the cell types, pathways, and molecules involved in innate memory in food animals. Until innate training and tolerance are better understood, caution is warranted to determine the immunological and metabolic costs and efficacy of protection to specific diseases. Within innate memory is the potential to reduce disease burden and antibiotic use in animal agriculture, and we feel this area of investigation represents one of the most exciting fields of study for a new generation of scientists.

## Author Contributions

KB, CL, and JM wrote and reviewed the manuscript.

## Conflict of Interest

CL and JM have received research funding from different companies to perform studies on in-feed β-glucan or yeast product immunomodulation in swine or cattle. Results from those studies are not provided within this review. The remaining author declares that the research was conducted in the absence of any commercial or financial relationships that could be construed as a potential conflict of interest.
